# Thyroid hormone status in Ghanaian patients with chronic kidney disease

**DOI:** 10.11604/pamj.2018.29.137.12992

**Published:** 2018-03-01

**Authors:** Nii Ayite Aryee, Emmanuel Ayitey Tagoe, Victor Anomah, Benjamin Arko-Boham, David Nana Adjei

**Affiliations:** 1 Department of Medical Biochemistry, School of Biomedical and Allied Health Sciences, College of Health Sciences, University of Ghana, Korle-Bu, Accra, Ghana; 2 Department of Medical Laboratory Sciences, School of Biomedical and Allied Health Sciences, College of Health Sciences, University of Ghana, Korle-Bu, Accra, Ghana

**Keywords:** Thyroid hormone status, CKD, Ghanaian, lipid profile, TSH, FT3, FT4

## Abstract

**Introduction:**

There is limited data on the prevalence of thyroid dysfunction in Ghanaian individuals with chronic kidney disease (CKD). Studies exploring the effect of thyroid hormones on renal function decline are also scanty. Unrecognized thyroid dysfunction in CKD may increase the burden of adverse health outcomes. The aim of this study was to determine thyroid hormone status and lipid profiles in patients with CKD attending the Renal Unit of the Korle-Bu Teaching Hospital.

**Methods:**

60 clinically euthyroid patients with CKD, and 65 clinically euthyroid subjects without CKD were recruited for this study. Estimation of effective glomerular filtration rate (eGFR) was done using the 4-variable Modification of Diet in Renal Disease (MDRD) formula with subsequent staging of CKD (stages 2-4). Collected venous blood samples from all study participants were analyzed for creatinine, free triiodothyronine (FT3), free thyroxine (FT4), thyroid stimulating hormone (TSH), total cholesterol (TC), high density lipoprotein (HDL) cholesterol, low density lipoprotein (LDL) and triglycerides (TG).

**Results:**

Levels of TC, HDL, LDL, and TSH levels did not differ significantly between the two study groups. However, TG, VLDL, FT3 and FT4 levels were significantly higher in CKD patients than in the control group. TC, TG, HDL, LDL, VLDL and TSH levels were not significantly different between stages of CKD in study subjects, although FT4 and FT3 levels were significantly different between all stages of CKD.

**Conclusion:**

Higher levels of FT3 and FT4 but not TSH, are associated with the incidence of CKD and eGFR decline in Ghanaian CKD patients.

## Introduction

Chronic Kidney Disease (CKD) is a disease spectrum characterized by progressive loss of renal function over a period of time. It is manifested by decreased kidney function that lasts longer than three months, as quantified by measured or estimated glomerular filtration rate [1]. A trend towards a global increase in CKD prevalence has been reported, leading to an acknowledgment of CKD as a public health problem [2]. Thyroid hormones have distinct effects on cellular growth and differentiation. They also modulate important physiological functions in virtually every human tissue [3]. Alterations in thyroid hormone status have been observed in patients with CKD, alongside the prevalence of thyroid dysfunction which has been shown to increase with progression of CKD [2,4]. Although the ability to detect subtle changes in the status of thyroid hormones has improved with the array of modern laboratory testing currently available, no recommendations are available regarding the treatment of mild abnormalities of thyroid hormone levels in patients with CKD not requiring dialysis. These mild abnormalities in thyroid hormone levels may represent risk factors for adverse health outcomes such as cardiovascular disease, and could be implicated in kidney disease progression [4]. There is accumulating evidence to suggest that derangement in lipoprotein metabolism may be a likely cause of kidney dysfunction [5,6]. Elevated TG levels and/or low high-density lipoprotein cholesterol (HDL-c) levels have been suggested to be predictive of an increased risk of renal dysfunction in a number of studies [5-7]. The progression of CKD has also been associated with the development of a number of complications, including dyslipidemia, thyroid dysfunction and cardiovascular disease [4]. Therefore, early identification and treatment of individuals with CKD who are at risk of adverse cardiovascular events is required to prolong survival and improve the quality of life for patients, particularly in developing countries where renal replacement therapy resources are limited, unaffordable or non-existent [8]. In a multi-center screening study in Ghana to identify the prevalence of CKD among hypertensives in an out-patient setting, an overall CKD prevalence of 46.9% was reported [9]. This was similar to findings from an earlier review of autopsy data [10]. No studies have however reported on altered thyroid hormone status in CKD in the Ghanaian setting. In view of the reported altered thyroid function and dyslipidemia observed in some CKD patients in other populations and the lack of data on thyroid status and lipid profile changes in the Ghanaian subjects with CKD, we undertook this study to determine the thyroid hormone status in Ghanaian patients with CKD and its association with lipid parameters.

## Methods

### Study design and recruitment of participants

This case control study was conducted over a 12 month period from July 2014 to June 2015 at the Renal Unit of the Korle-Bu Teaching Hospital (KBTH), Korle-Bu, Accra. KBTH is the leading national referral center in Ghana and the only tertiary health facility in the southern part of Ghana. There are clinical services for both adult and pediatric renal patients. 60 non-dialysis CKD patients of different CKD stages (stages 2-4) with no previous history of thyroid dysfunction accessing services at the Renal Unit of the KBTH and 65 apparently healthy control subjects residing in the nearby communities surrounding KBTH were recruited for this study. All recruited patients with non-dialysis dependent CKD stages 2-4 were assessed for thyroid function. Informed consent was obtained from all study participants prior to their inclusion in this study. CKD patients were made to understand that opting out from this study would not affect their medical care. A questionnaire was administered to each patient at recruitment to obtain demographic and anthropometric data, as well as full clinical history including diabetes status and polycystic kidney disease. At the assessment, a dipstick urinalysis was also performed. Blood pressure (BP) was measured by first allowing the study participant to rest for 15 minutes prior to measurement.

### Case definition

Chronic kidney disease (CKD) was defined as the occurrence of kidney damage or decreased kidney function manifested by abnormal albumin excretion and quantified by measured estimated glomerular filtration rate (eGFR) of ≤90 mL/min that persisted for more than three months with proteinuria. The eGFR was calculated using the abbreviated Modification of Diet in Renal Disease (MDRD) study formula [**1**]. Proteinuria was defined as a dipstick reading of trace or 1+, or heavy (urine dipstick reading greater than or equal to 2+). Stages of non-dialysis-dependent CKD (stages 2-4), were defined according to the American National Kidney Foundation as follows: Stage 2: eGFR 60-89 ml/min/1.73 m^2^, stage 3: eGFR 30-59 ml/min/1.73m^2^ and stage 4,15-29 ml/min/1.73 m^2^ [**1**].

### Blood sample collection and laboratory procedure

Participants consented by endorsing a written consent form before samples were collected. 5ml venous fasting blood sample was collected from each participant and dispensed into different sample tubes. Samples in plain tubes were processed for total cholesterol (TC), triglycerides (TG), high density lipoprotein cholesterol (HDL-c), VLDL, serum creatinine, TSH, FT3 and FT4 estimation. Thyroid hormone Assay (TSH, FT3 and FT4) kits, purchased from CentronicGMbH (Kleinfeld, Germany) were used to assay TC, TG and serum creatinine. HDL-c was determined using kits from Biosystems (Barcelona, Spain). The manufacturer's protocol was followed for all tests using Vital Scientific Microlab 300M (VSM 300) automated analyzer. LDL-c was calculated using Friedwald equation [11]. Quantification of thyroid hormones was determined by enzyme-linked immunosorbent assay (ELISA), (Human, Germany), and Labsystems Multiscan-352 plate reader (Finland). All study participants were categorized into various thyroid function groups based on results of thyroid function tests. Thyroid dysfunction was considered if a participant's thyroid hormone level fell outside the reference range (TSH normal range: 0.4-5.5 μIU/ml), FT3 normal range: 2.8-7.3pmol/L, FT4 normal range: 8.5-22.5 pmol/L). For example, subclinical hypothyroidism was defined as TSH levels greater than 5.5 mIU/ml, with FT3 and FT4 levels within the normal range. Participants who at baseline had a history of thyroid disease or use of thyroid medications, history of cancer and type 2 diabetes mellitus (D2M) were excluded.

### Ethical consideration

institutional ethical clearance and approval for the conduct of the study was given by the ethical and protocol review committee of the College of Health Sciences (CHS), Korle-Bu.

### Statistical analysis

Continuous data were presented as mean and standard deviation (mean ± SD). Student t-test was used to compare two group means and ANOVA for three or more group means using Graphpad Prism 5.0. Hypertension prevalence, CKD stages and thyroid dysfunction groups were expressed in percentages.

## Results

### Demographic and clinical parameters

125 participants who met the inclusion criteria were recruited for this study. Clinical parameters and demographic data of the study population are presented in Table 1. There were significantly more females in the control group than in the CKD patient group. The mean age of the CKD patients was higher than that of the control group, but not significantly different between the two study groups. Body mass index was statistically different between the two study groups. Blood pressure was significantly higher in CKD patients when compared to the control group. The prevalence of hypertension in the CKD study group was 75%.

**Table 1:** Demographic and clinical parameters of the study population

Parameter	CKD patients(N = 60)	Control(N = 65)	95% CIof mean diff	p-value
Age (yrs)	51.83 ± 16.58	45.52 ± 11.25	1.14-11.48	0.017*
Females	23% (47)	39% (60)		< 0.05*^Y^
BMI (kg/m^2^)	27.80 ± 4.86	26.13 ± 6.30	-0.48-3.81	0.127
SBP (mmHg)	136.94 ± 23.29	117.51 ± 15.58	12.21-26.65	< 0.001*
DBP (mmHg)	83.00 ± 14.41	75.86 ± 12.63	2.09-12.15	0.006*
Hypertension (%)	75.0			

N = sample size, CI= confidence interval, BMI = body mass index, SBP = systolic blood pressure, DBP = diastolic blood pressure, D2M = type 2 diabetes mellitus.

Y= Z- scores for proportion comparison. Result is presented as mean ± standard deviation. *P-values less than 0.05 were considered statistically significant

### Biochemical parameters

Comparison of biochemical parameters of CKD patients and the control group is presented in Table 2. Creatinine levels were significantly higher in the CKD group than in the control group. Creatinine and estimated glomerular filtration rate (eGFR) were significantly reduced in the CKD group when compared to the control group. Baseline biochemical parameters for total cholesterol (TC), HDL, LDL-c and TSH levels did not differ significantly between the two study groups. However, TG, VLDL, FT3 and FT4 levels were significantly higher in the CKD group.

**Table 2:** Biochemical and thyroid hormones levels of the studied population

Parameter	CKD patients(N = 60)	Control(N = 65)	95% CIof mean diff	p-value
Serum Creat. (mmol/L)	189.84 ± 111.42	78.06 ± 20.59	83.81 - 139.73	< 0.001*
Creat. Clear. (ml/min)	55.33 ± 38.29	107.12 ± 43.54	- 67.29 - (-36.27)	< 0.001*
GFR (ml/min/1.73m^2^)	63.83 ± 46.63	132.78 ± 35.09	- 84.12 - (-53.80 )	< 0.001*
TC (mmol/l)	4.74 ± 1.78	4.98 ± 1.09	-0.77 - 0.30	0.001*
TG (mmol/l)	1.60 ± 0.88	1.09 ± .72	0.22 - 0.79	0.001*
HDL-c (mmol/l)	1.19 ± 0.22	1.14 ± 0.19	-0.02 - 0.13	0.158
LDL-c (mmol/l)	3.12 ± 2.33	3.3 ± 0.84	-0.84 - 0.39	0.471
VLDL-c (mmol/l)	0.74 ± 0.50	0.50 ± 0.33	0.10 - 0.37	0.001*
TSH (mIU/ml)	1.87 ± 1.51	2.36 ± 1.31	-1.02 - 0.03	0.064
FT3 (pmol/L)	4.83 ± 2.72	3.43 ± 0.70	0.70 - 2.09	< 0.001*
FT4 (pmol/L)	14.32 ± 2.91	10.80 ± 2.71	2.47 - 4.57	< 0.001*

N = Sample size, CI = Confidence Interval, CKD = Chronic Kidney Disease, Serum Creat = Serum Creatinine, Creat. Clear. = Creatinine Clearance, GFR = Glomerular Filtration Rate, TC = Total Cholesterol, TG = Triglyceride, HDL-c = High Density Lipoprotein cholesterol, LDL-c = Low Density Lipoprotein cholesterol, VLDL-c = Very Low Density Lipoprotein cholesterol, TSH = Thyroid stimulating hormone, FT3 = Free triiodothyronine, FT4 = Thyroxine Result is presented as mean ± standard deviation.

* P-values less than 0.05 were considered statistically significant.

### Biochemical parameters and thyroid hormone profile stratified by CKD stage

Various biochemical parameters and thyroid hormone profiles stratified by CKD stage are presented in Table 3. CKD participants in stages 3 and 4 were significantly older than those in stages 1 and 2. Serum creatinine levels were significantly different between all stages of CKD and showed an increasing trend from stage 1 to stage 4, whereas creatinine clearance decreased significantly across CKD stages 1-4 (p < 0.001). Levels of TC, TG, HDL-c, LDL-c, VLDL and TSH were not significantly different between the stages of CKD in our study subjects. FT4 and FT3 levels were significantly different between all the stages of CKD. However, FT4 levels showed greater variation between CKD stages than FT3.

**Table 3:** Clinical, biochemical and thyroid hormonal status of chronic kidney disease stages

Parameter	Chronic kidney disease stages				p-value
	Stage 1(n= 13)	Stage 2(n = 7)	Stage 3(n = 13)	Stage 4(n = 16)
Age (years)	41.61 ± 17.03	45.57 ± 14.73	58.69 ± 1.18	57.31 ± 10.73	0.015*
Sr.Cr (mmol/L)	74.77 ± 14.62	113.86 ± 16.16	193.46 ± 49.75	313.63± 83.35	0.000*
Cr.Cl. (ml/min)	107.88 ± 29.83	64.94 ± 15.75	36.91 ± 7.22	23.42 ± 4.71	0.000*
eGFR(ml/min/1.73m^2^)	129.20 ± 33.22	75.01 ± 9.89	43.66 ± 8.27	22.20 ± 4.25	0.000*
T.chol (mmol/l)	5.02 ± 1.42	3.91 ± 1.38	4.76 ± 2.21	4.86 ± 1.85	0.296
TG (mmol/l)	1.74 ± 1.09	1.44 ± 0.72	1.63 ± 1.11	1.52 ± 0.77	0.234
HDL-c (mmol/l)	1.22 ± 0.16	1.09 ± 0.13	1.22 ± 0.27	1.19 ± 0.25	0.491
LDL-c (mmol/l)	3.00 ± 1.32	2.17 ± 1.03	3.61 ± 1.31	3.24 ± 1.92	0.304
VLDL-c (mmol/l)	0.81 ± 0.50	0.66 ± 0.31	0.76 ± 0.50	0.69 ± 0.35	0.215
TSH (mIU/ml)	1.08 ± 0.76	2.03 ± 1.36	1.65 ± 0.77	2.60 ± 0.88	0.354
FT3 (pmol/L)	5.38 ± 2.34	6.19 ± 4.71	4.45 ± 2.49	4.09 ± 1.88	0.013*
FT4 (pmol/L)	14.00 ± 3.51	15.51 ± 3.82	14.64 ± 0.98	13.80 ± 3.09	0.000*

n = sub-group size, CKD stage definition: Stage 2 = 60 ≤ eGFR ≤ 89 ml/min/1.73m^2,^ Stage 3 = 30 ≤ eGFR ≤ 59 ml/min/1.73m^2,^ Stage 4 = 15 ≤ eGFR ≤ 29 ml/min/1.73m^2.^

* P-values less than 0.05 were considered statistically significant

### Thyroid function status

Results of thyroid function status for our study groups are shown in Table 4. Subclinical hypothyroidism was observed in 2% and 3 % of the CKD patient group and non-CKD group respectively whereas 2% of CKD patients were diagnosed with subclinical hyperthyroidism.

**Table 4:** Thyroid hormone dysfunction prevalence in the studied population

Group / Thyroid disorder	**CKD patients(N = 49)**	**Non-CKD Control(N = 65)**
Sub-clinical hypothyroidism n(%)	2(3.3 )	3 (4.6)
Primary hypothyroidism n(%)	0(0.0)	0(0.0)
Sub-clinical hyperthyroidism n(%)	2(3.3)	0(0.0)
Primary hyperthyroidism n(%)	0(0.0)	0(0.0)

TSH normal range: 0.4 - 5.5 mIU/ml, FT3 normal range: 2.8 - 7.3pmol/L, FT4 normal range: 8.5 - 22.5 pmol/L. Subclinical Hypothyroidism: when TSH is higher than 5.5 mIU/ml, FT3 and FT4 within normal range. Primary Hypothyroidism: when TSH is greater than 5.5mIU/ml, FT3 and FT4 less than normal. Subclinical Hyperthyroidism: when TSH is less than 0.3 mIU/ml, FT3 and FT4 within normal range. Primary Hyperthyroidism: when TSH is less than 0.3mIU/ml, FT3 and FT4 higher than normal

## Discussion

The impact of thyroid dysfunction on renal function is linked to the role of the kidneys in metabolism, degradation and excretion of several substances including thyroid hormones. It is therefore expected that any impairment in kidney function could lead to altered thyroid physiology [2]. Currently there is no report on the thyroid status of clinically Ghanaian euthyroid CKD patients not requiring dialysis. We demonstrated in this study that TSH levels did not differ significantly between CKD patients and clinically euthyroid participants without CKD. Furthermore, FT3 and FT4 levels were found to be significantly higher in CKD patients than in the control group, although mean FT3 and FT4 values, as well as that of TSH were all within the reference range. Similarity in the levels of TSH in the control group and CKD subjects has been observed in some studies that focused on thyroid function in end stage renal disease [12, 13]. Other studies, mainly in Asian and Caucasian populations have shown that serum TSH levels are usually normal or elevated in CKD patients with normal or low free and total T3 and T4 levels [14, 15]. Our results, particularly for serum FT3 and FT4 levels in CKD patients, though at variance with studies that have reported a low T3 and T4 as the most frequently observed thyroid alteration in CKD patients, still supports the growing body of evidence that thyroid hormone alterations occur in CKD individuals not requiring dialysis. Elevated FT3 and FT4 levels observed in our CKD participants may have been confounded by concomitant hypertension. Numerous studies have identified a linkage between thyroid dysfunction and hypertension, showing that both hyperthyroidism (overactive thyroid) and hypothyroidism (underactive thyroid) are associated with elevated blood pressure [16]. Milder forms of thyroid dysfunction such as subclinical hyper- and hypothyroidism, it has been suggested, may raise also the risk for high blood pressure [17]. Several possible mechanisms have been used to explain the link between thyroid hormones and renal function. In overt hypothyroidism, cardiac output is decreased and systemic vascular resistance is increased, resulting in decreased renal blood flow, decreased GFR and creatinine clearance and increased serum creatinine [16, 17].

Conversely, cardiac output and circulating blood volume are increased in hyperthyroidism, resulting in increased creatinine clearance and decreased serum creatinine [16]. Although these mechanisms have been used to explain the link between thyroid dysfunction and hypertension, their role in euthyroid individuals with low to normal thyroid function is unclear [4]. We could neither find a significant increasing or decreasing trend for TSH across CKD stages. Although FT3 and FT4 levels were significantly different across CKD stages 2-4, we could not ascertain an increasing or decreasing trend for these thyroid hormones. Our findings were different from results of the Kangbuk Samsung Health Study (KSHS), performed among 104,633 Korean participants with an average age of 38.0 years, and normal thyroid function at baseline. It reported that high-normal levels of TSH and low-normal FT3 levels, but not FT4 levels, were associated with increased risk of incident CKD, defined as eGFR<60 mL/min/1.73 m^2^ [18]. The disparate definitions of CKD, as well as the relatively older individuals participating in our study compared to those in KSHS study, might possibly account for this discrepancy. Studies have also described the expression of TSH receptors in extra-thyroidal tissues including the kidney. It is therefore plausible that TSH may affect renal function independently of FT4 or FT3 [17]. Only 2% and 3% of the CKD and control group respectively showed laboratory evidence of clinical hypothyroidism whereas 2% of CKD patient group were diagnosed with subclinical hyperthyroidism. It is noteworthy to add that evidence of endocrine dysfunction commonly consists of laboratory abnormalities, many of which are not associated with apparent disease. Our study had a number of limitations. The small sample size limited our ability to determine significant thyroid dysfunction in the study population in the absence of clinical disease. Secondly, we did not determine levels of anti-thyroid peroxidase antibodies. Autoimmune thyroid disease may lead to the deposition of thyroglobulin- containing immune-complexes in glomeruli, which can cause glomerular injury in autoimmune thyroiditis [19]. In future studies, we may have to consider different or additional criteria for calculating eGFR that will have less bias and introduce higher accuracy than the Modification of Diet in Renal Disease Study equation, especially at higher GFR levels [20].

## Conclusion

We found that higher levels of FT3 and FT4, but not low levels of TSH, to be associated with incident CKD and an eGFR decline. To improve upon the biochemical criteria for diagnosing thyroid dysfunction, additional criteria for calculating eGFR and larger sample size may be required in assessment of thyroid hormone status.

### What is known about this topic

- Thyroid dysfunction causes significant changes in kidney function and kidney diseases can be associated with thyroid disorders;
- Alterations in Thyroid hormone status and dyslipidemia are common disorders in patients with CKD.

### What this study adds

- The equation developed from the MDRD study may not provide a more accurate estimation of GFR in our study group;
- Our findings highlight the importance of regular screening for thyroid dysfunction in patients with CKD using improved biochemical criteria for diagnosing thyroid dysfunction.
